# The Development of the CAIRDE General Awareness Training

**DOI:** 10.3390/ijerph22081306

**Published:** 2025-08-20

**Authors:** Jack Sweeney, Noel Richardson, Paula Carroll, P. J. White, Emilie Roche, Shane O’Donnell

**Affiliations:** The National Centre for Men’s Health, Department of Health and Sports Sciences, South East Technological University, Kilkenny Road Campus, Kilkenny Road, R93 V960 Carlow, Ireland; c00277225@setu.ie (J.S.); paula.carroll@setu.ie (P.C.); pj.white@setu.ie (P.J.W.); emilie.roche@postgrad.setu.ie (E.R.); shane.odonnell3@hse.ie (S.O.)

**Keywords:** suicide, mental health, intervention development, construction, occupational health training, suicide prevention

## Abstract

Suicide is a leading cause of death among construction workers, particularly younger and lower-skilled employees. Barriers such as stigma, low mental health literacy, and traditional masculine norms hinder help-seeking in this male-dominated sector. Few mental health interventions are tailored to this context. This study developed a co-designed, theory-informed training to improve mental health literacy, reduce stigma, and increase help-seeking among construction workers in Ireland. Using the Medical Research Council’s framework, the training was developed with the Theory of Planned Behavior (TPB), Behavior Change Techniques, and extensive stakeholder co-design. Two systematic reviews, a broad literature review, and focus groups with industry managers informed the content and structure. The training will be pilot-tested using validated measures: the Literacy of Suicide Scale (LOSS), the Stigma of Suicide Scale (SOSS), and the General Help-Seeking Questionnaire (GHSQ), the results of which will be the subject of a separate study. CAIRDE is a promising, evidence-based training that addresses key mental health barriers in Irish construction. Embedding the TPB within a co-design methodology has resulted in the development of a training program that is underpinned by theoretical fidelity and cultural relevance and provides a framework for other male-dominated industries to draw upon. Future work should address remaining challenges related to stigma and help-seeking, and explore broader implementation through integration into mandatory safety training.

## 1. Introduction

Internationally, suicide is the leading cause of death among construction workers. Extensive research over the past decade has revealed disproportionately high suicide rates within the construction industry worldwide [1,2,3,4]. Notably, lower-skilled workers [3,5] and younger construction workers [2] are at greater risk than their counterparts. Key risk factors associated with suicide in the construction sector include depression, long working hours, financial stress caused by low-income levels, job insecurity, unrealistic job expectations, substance use (including drug/opioid use), alcohol abuse, poor physical health, and suicide bereavement [2,4,6,7,8,9]. Suicide risk within the industry is compounded by an industry culture that upholds more traditional masculine beliefs, such as self-reliance and stoicism. This serves as a barrier to emotional expression and help-seeking [10,11] and, in common with other male-dominated industries, further exacerbates and perpetuates mental health issues such as psychological distress [12] and depression [13], and increases the risk of suicide [14]. The construction industry in Ireland comprises a workforce of 170,000 employees, with a large proportion of foreign workers, working predominantly in small construction companies. The occupation often involves prolonged periods away from family and friends, long working hours, and long commute times. This, combined with the culture embedded in the industry, has resulted in an estimated 10% of all suicides in Ireland in 2019 being by construction workers. These findings underscore the urgent need for targeted mental health interventions and support systems within the construction industry to address and mitigate these risks.

Despite the significant mental health burden faced by construction workers, challenges related to mental health literacy, help-seeking behaviors, and stigma remain pervasive in this population. Mental health literacy tends to be lower among males compared to females [15], and older men typically exhibit lower levels of mental health literacy than their younger counterparts [16]. Notably, this contrasts with findings that suggest older construction workers demonstrate higher levels of suicide prevention literacy compared to younger workers [17]. Stigma surrounding mental health remains widespread across the industry, posing a substantial barrier to both help-seeking and help-offering behaviors [18].

Tailored training programs focused on mental health and suicide prevention have proven effective in reducing suicide rates within the general population [19,20,21]. However, the development and refinement of such training programs to meet the unique needs of men in male-dominated industries—such as construction—has been limited. While initiatives like MATES in Construction (Australia) have shown potential and have shown evidence of being effective [22], they lack a robust theoretical underpinning in terms of tracking any potential behavior change mechanisms that may be at play. Nevertheless, these programs provide a useful foundation for developing an intervention that not only accounts for the cultural and social norms specific to the Irish construction sector but is also grounded in a deeper theoretical understanding of the behavioral mechanisms that are at play. Embedding behavior change concepts into the design of a targeted mental health and suicide prevention training program—alongside the active involvement of stakeholders at all levels, from laborers to CEOs—facilitates the development of an intervention that is both acceptable and feasible within the industry. Combining this with existing models, such as Joiner’s Interpersonal–Psychological Theory of Suicide (IPST) and the Stress-Diathesis Model, and educating the construction workers on the extensive risk factors associated with the profession, may help instill change.

A systematic review of interventions based on the Theory of Planned Behavior (TPB) identified attitudes and perceived behavioral control as the most influential factors in shaping individuals’ intention to seek help [23]. Previous studies have successfully utilized behavior change theories in combination with co-design workshops to create interventions across a wide range of health care settings, including geriatric drug use reduction in hospital care [24], the development of online resources for dementia [25], initiatives addressing drug use in maternity care [26], and recycling uptake within hospitals [27]. Embedding the TPB within co-design workshops allows for the extraction of key constructs—attitudes, subjective norms, and perceived behavioral control—while also gathering rich, qualitative insights through collaborative engagement. This provides a strong platform and evidence base for developing a mental health and suicide prevention intervention that is grounded in behavior change theories. The study addresses the paucity of theoretically grounded, culturally relevant suicide prevention programs for the construction industry. We hypothesize that an intervention informed by behavior change theory and tailored to industry culture through co-design will enhance mental health and suicide literacy, help-seeking, and help offering among construction workers, along with decreasing mental health and suicide stigma among construction workers. As such, this paper outlines the development and validation of a co-designed and theory-informed general awareness training (GAT) program aimed at improving mental health literacy with a specific focus on suicide literacy, reducing both public and self-stigma of mental health and suicide, and enhancing both help-seeking and help-offering intentions among construction workers in Ireland. This was part of a wider project entitled ‘CAIRDE’ (Irish word for ‘friends’ **C**onstruction **A**ll**i**ance to **R**educe Suici**de**), which was commissioned by the National Office for Suicide Prevention in Ireland to reduce suicide stigma, enhance knowledge of suicide and mental health issues, and increase help-seeking and help-offering in the construction industry.

## 2. Materials and Methods

### 2.1. Development Framework

The Medical Research Council’s (MRC) framework for developing a complex intervention guided the development of the program. This was supplemented by behavior change theory—the Theory of Planned Behavior (TPB) and behavior change techniques taxonomy (BCTTs) derived from the Behavior Change Wheel (BCW)—and underpinned by a co-design methodology. This theoretical and participatory approach was selected to ensure that the intervention would be both evidence-based and contextually appropriate for the unique cultural and occupational environment of the construction industry in Ireland.

**Figure 1:**
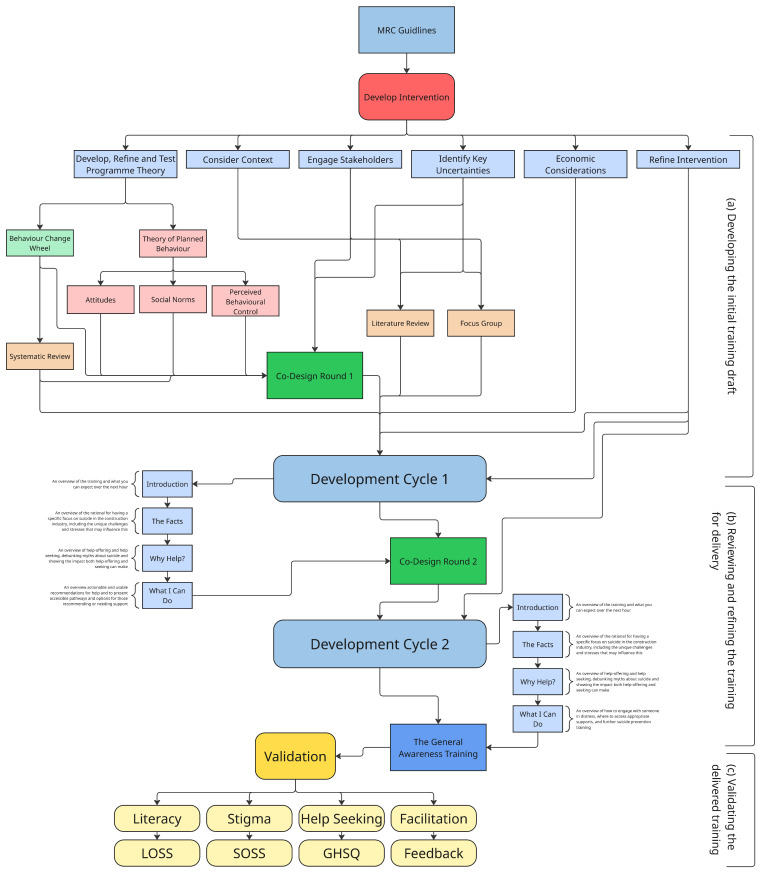
Process and theory map.

The MRC framework divides complex intervention development into four phases—development or identification of the intervention, feasibility, evaluation, and implementation—with each phase being underpinned by a common set of core elements (see Figure 1). Intervention development can apply to the development of a new intervention or adapting an existing intervention for a new context, based on research evidence and theory. Feasibility refers to the assessment of the feasibility and acceptability of the intervention and evaluation design in order to make decisions that progress to the next stage of evaluation. Evaluation refers to the assessment of an intervention using the most appropriate methods to address the research question. Implementation refers to the deliberate efforts to increase the impact and uptake of successfully tested health innovations [28].

The focus of the current study is on program development and feasibility. The TPB was used to explore the key psychosocial determinants of help-seeking and help-offering behaviors: attitudes, subjective norms, and perceived behavioral control. These constructs have been consistently shown to influence intention and behavior in health-related contexts, including mental health [23,29]. The BCW was used to explore the underlying behavior change techniques and methodologies utilized in previous interventions targeting men’s mental health components [30,31], and to apply those that showed effectiveness into a new intervention designed specifically for the Irish construction industry. Co-design was employed to ensure the intervention was grounded in the lived experiences of those within the construction industry.

A multi-step process involving stakeholder engagement, systematic reviews, and iterative co-design cycles was undertaken to ensure contextual relevance and theoretical fidelity, thus improving the acceptability and feasibility of a training program that sought to engage a ‘hard to reach’ (ref) population group. This development cycle for the training is outlined in Figure 1. An initial draft of the training program was developed based upon a review of existing evidence, the outcomes from focus groups conducted with industry stakeholders [32] and the co-design exploration and development of TPB components around each of the mental health outcomes being targeted (mental health and suicide literacy, mental health and suicide stigma, and help seeking behaviors. These workshops were conducted at two key stages: during the initial development phase (R1) and after a draft intervention had been created (R2), allowing for more refined discussion, critique, and improvement of the program. The co-design workshops created an inclusive space where construction workers from different trades could participate equally with mental health professionals, intervention facilitators, and the researchers developing the intervention. The evaluation will be integrated into the pilot training delivery in the form of a pre- and post-intervention quasi-experimental study to assess the initial validity of the intervention. The set of validated instruments will include the Literacy of Suicide Scale (LOSS) to measure mental health literacy, the Stigma of Suicide Scale (SOSS) to assess stigma, and the General Help-Seeking Questionnaire (GHSQ) to evaluate help-seeking intentions. Consent from each participant was obtained, and data was collected pre- and post-intervention to gauge changes in the key outcomes. The integration of each theoretical underpinning is explored through the MRC framework below. Ethical approval was granted by South East Technological University’s ethics committee (Reference Number #313).

### 2.2. Identifying the Evidence Base

The first key step to developing complex interventions is to explore and synthesize what is already known about similar interventions and the methods that have been used to evaluate them [33,34]. As such, systematic reviews of similar interventions must be evaluated, and if none such exist, then a systematic review of high quality should be conducted. The evidence base for previous interventions in male-specific and male-dominated workplaces was limited at the time of development, primarily focusing on signs of and risk factors for suicide [35] and lifestyle interventions for men’s mental health [36]. No systematic reviews had been conducted on interventions targeting stigma towards men’s mental health or on workplace interventions targeting mental health literacy, stigma, help-seeking, or help-offering in male-dominated industries. In response to this, two systematic reviews investigating these components were conducted [30,31]. Sweeney et al. (2024) [31] systematically reviewed interventions specifically targeting men’s mental health stigma, identifying eleven interventions that predominantly utilized psychoeducation, social contact, and credible male role models to address both public and self-stigma. The findings suggested that the most effective interventions combined educational content with therapeutic elements that encouraged men to reframe unhelpful thoughts and directly challenge restrictive masculine norms, with the involvement of respected male figures and open dialog proving particularly impactful. In parallel, Roche et al. (2024) [30] reviewed workplace interventions in male-dominated industries. They found that psychoeducational resources, skills training, and peer support mechanisms were most successful in improving mental health literacy and intentions to seek help, although evidence for sustained behavioral change and stigma reduction was more limited. Both reviews highlighted the critical importance of tailoring interventions to the cultural context of masculinity, employing behavior change techniques such as providing information, modeling, and active engagement, while also noting persistent methodological limitations like inconsistent outcome measures and variable and inconsistent use of theory. Together, these reviews underscore the value of combining credible role models, targeted psychoeducation, and explicit engagement with masculine norms in the design of interventions. They also emphasize the need for rigorous, theory-driven development and evaluation to ensure these approaches are both effective and replicable. This informed both the development and evaluation of the GAT intervention explored in this paper.

### 2.3. The Stepwise Development Process

As per Figure 1 above, the intervention was developed via three distinct cycles that included (a) developing an initial training draft, (b) reviewing and refining the training for delivery, and (c) validating the delivered training. The first and second of these will be described in detail here; a summary of the validated evaluation tools will also be provided, but the outcomes of the evaluation will be the focus of a follow-up paper.

#### 2.3.1. Development Cycle 1: Design of the Initial Training Draft

The development of an initial training draft included developing program theory, considering context, engaging stakeholders, and identifying key uncertainties. Although some consideration was given to economic aspects, the primary focus in this initial pilot phase was on the acceptability and feasibility of the training. Economic considerations will be fully addressed in the next stage of development. All of the information gained through these mediums was collated and synthesized by the research team and integrated into a series of co-design workshops (Round 1; R1) to produce a draft training program. The training design was guided by existing models such as the ENGAGE suite of men’s health training programs in Ireland and shaped to reflect the daily realities of construction workers. The process of developing this first training draft is detailed in the following sections.

#### 2.3.2. Develop Program Theory

Factors impacting help-seeking among men and construction workers specifically include themes around level of acceptance of help-seeking by peers, personal challenges, cultural and environmental influences, self-medication, perspectives on seeking professional help, and traditional masculine ideals [37]. These factors can act not only as a psychological or physical barrier to help-seeking but can also reduce the person’s motivation or intention to do so in the first place. As such, the program theory was developed using the TPB, proposing that changes in attitudes, perceived social norms, and perceived behavioral control would influence intentions to seek and/or offer help for mental health concerns, while also contributing to the destigmatizing of mental health. The destigmatisation was also targeted using the findings from the previous systematic reviews conducted as part of the wider project [30,31].

Each component of the TPB, as it relates to help-seeking and mental health stigma, was incorporated into the first draft of a co-design workshop, utilizing each component as a section of the workshop. These components were explored alongside relevant behavior change techniques and functions identified in the aforementioned systematic reviews. Each section of the co-design workshop was structured around a specific component of the TPB (see Table 1). The development of the training across the two development cycles is detailed in Table 1.

#### 2.3.3. Consider Context

The construction industry presents unique challenges to mental health promotion, including a predominantly male workforce, a culture of stoicism, transient employment patterns, and variable work conditions [18]. Previous interventions developed in this area, typically in either Australia or America, have achieved some success in targeting specific factors associated with suicidality among construction workers [22]. This training was designed as a universal training for all construction workers and was developed in tandem with a more in-depth program supporting construction industry managers with direct intervention (Connector Training). This tiered approach was intended to have the broadest possible engagement. As per Figure 1 above, context in terms of cultural considerations, along with the social and practical implications of such a training program, were explored during the first set of co-design workshops (see below) and informed by focus groups (*n* = 5) conducted as part of the wider project [32]. These engagements provided rich insights into the day-to-day experiences of construction workers, including their struggles with mental health. They highlighted workers’ unique potential to act as sources of support to their fellow workers within the construction industry, as they spend extended periods on site with their peers. They also stressed the importance of fostering an open culture around mental health on-site, understanding the practice of help-offering, and using the right skills, language, and resources to help a colleague in distress. Models such as the IPTS help explore the risk factors associated with the construction industry, unifying through mutual understanding. The context was also informed by two systematic reviews [30,31] as well as a review of other relevant literature. The intervention also built upon the experience gained and lessons learned from men’s health policy implementation in Ireland [38]; specifically the development and implementation of national men’s health training program (ENGAGE) which has shown positive impact on service providers capacity to engage and work with men [38], as well as the development of new training programs targeting the upskilling of key stakeholders to engage men in workplace settings (e.g., farming, [39]).

#### 2.3.4. Engage Stakeholders and Identify Uncertainties

Stakeholder engagement was prioritized throughout the development process. In this cycle of training development, stakeholder engagement happened via focus groups and the R1 Co-design workshops (see below). Regarding the former, as part of the wider CAIRDE project, Roche et al. (2025) [32] explored the experiences of construction managers in offering help to workers within the context of a male-dominated construction industry, focusing on the cultural and organizational factors that shape help-offering behaviors. The study found that managers recognized the importance of normalizing conversations about mental health and help-seeking, emphasizing that consistent, everyday actions and open dialog were necessary to make help-offering a routine part of workplace culture. Key enablers included strong relationships, trust, and the modeling of supportive behaviors by leaders. Conversely, barriers involved stigma, concerns about appearing weak, and the pressures of a demanding work environment. The findings demonstrate that effective help-offering in construction relies on leadership that actively challenges stigma, integrates support into daily practices, and fosters an environment where seeking and offering help are seen as a normal and valued aspect of the job.

Key uncertainties related to intervention delivery, content relevance, and engagement strategies. These issues were identified through the systematic reviews, a wider trawl of the literature, the co-design workshops (R1), and focus group discussions. Addressing these uncertainties—guided by the frameworks provided by the TPB and the MRC—was essential for refining the program theory and ensuring alignment with the specific needs of construction workers in Ireland.

#### 2.3.5. Co-Design Round 1

**Figure 2:**
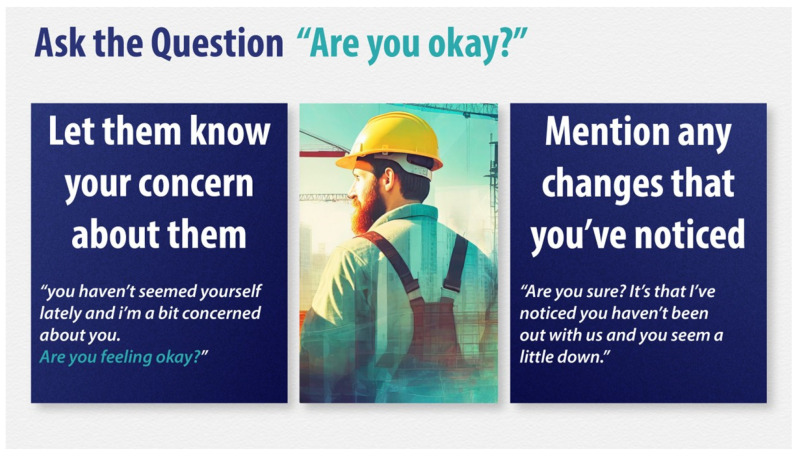
Visual media example.

**Figure 3:**
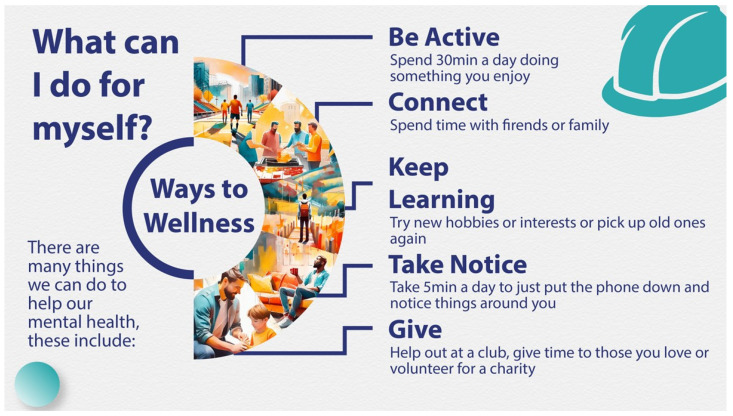
Visual media example 2.

The next phase of the initial draft program development comprised three co-design workshops (R1) involving seventeen participants (fourteen male and three female): Workshop 1 (*n* = 7), Workshop 2 (*n* = 3), and Workshop 3 (*n* = 7). Participants were recruited from three large construction sites across Ireland and represented a wide range of professional roles, from office cleaner to site foreman, to ensure broad stakeholder engagement. Participants ranged in age from 18 to 65 years, with industry experience ranging from less than one year to 47 years. The workshops were held either in site offices or training rooms and were conducted during regular working hours. Each workshop focused on a detailed exploration of the TPB components around each of the mental health outcomes being targeted. These sessions aimed to gather insights into the everyday lived experience of those working in the industry, including what they perceived as the priority needs that the training should address. By involving these diverse voices and perspectives, it was anticipated that the intervention would not only be made more relevant and accessible but also more likely to be accepted and adopted within the industry. The workshops provided critical feedback on language, cultural relevance, practical delivery, and emotional resonance. This feedback also informed the creation of specific training resources (see Table 1). Visual media and narratives were developed to authentically represent construction workers’ experiences (see Figure 2 and Figure 3). A key element was the development of a video narrative based on the collective experiences of participants in the co-design workshops. This served both as a narrative persona onto which participants could reflect and contextualize their own life experiences, enhancing emotional engagement and material relevance. Overall, these co-design workshops gave construction workers a direct voice in shaping the intervention, ensuring its relevance, acceptability, and feasibility within the context of the Irish construction industry. A draft of the training program was developed by the end of Development Cycle 1, with the consensus from R1 workshops being that it should be no more than one hour in duration.

### 2.4. Development Cycle 2: Reviewing and Refining the Training for Delivery

The purpose of this phase of program development was to consult with relevant industry, professional, and training experts to make final adjustments and refinements to the program (co-design R2), as well as to map it to behavior change theory.

#### Co-Design Round 2

Following the development of the initial training draft, a final co-design workshop (R2) was conducted with industry experts. Thirteen participants (eight male, five female) took part, comprising a diverse range of expertise, including professional training facilitators, men’s health advocates, researchers specializing in men’s health, and representatives from three construction companies across roles such as laboring, management, health and safety, and occupational health. Participants’ ages again ranged from 18 to 65 years, with experience in the industry ranging from less than three years to 47 years. This workshop was conducted in a conference venue in central Dublin. The goal was to review and refine the intervention materials. The session focused on identifying content that might not be culturally relevant, unsuitable for a one-hour format, or lacking clarity. Feedback was used to further tailor the materials and adjust the delivery style, ensuring that the training was engaging, appropriate, and achievable within the time constraints.

Informed by the outcomes of the second co-design workshop, the training was iteratively refined, resulting in a revised final General Awareness Training (GAT) package. This version incorporated both stakeholder feedback and theoretical principles, ensuring that it was both practical and aligned with behavior change theory. Key refinements included reducing the perceived responsibility and pressure on participants to directly intervene in suicide-related situations—acknowledging that a more in-depth intervention would require additional time beyond the one-hour format. Instead, the training focused on awareness, support, and signposting to appropriate services. Agreement was also reached during this phase on the final visual and presentation style of the training materials, designed to reflect the lived experiences and communication preferences of construction workers.

## 3. Results

### 3.1. Modeling Processing and Outcomes

To ensure the training program systematically addressed the determinants of help-seeking and mental health stigma among men in the Irish construction industry, all content was mapped to the TPB framework. This involved aligning each section of the training with the TPB’s core constructs—attitudes, social norms, and perceived behavioral control—by identifying key uncertainties and barriers (e.g., stigma, recognition of risk factors, perceived value of help, and awareness of services) and directly linking these to targeted training components. The mapping process was iterative and informed by co-design workshops, where feedback from stakeholders was used to refine both the content and its theoretical alignment.

In parallel, Behavior Change Techniques (BCTs) were systematically identified and incorporated into the program content to operationalize the TPB constructs. For example, to address attitudes, BCTs such as providing information about health consequences, dispelling myths, and modeling positive behaviors were embedded in sections on illness recognition, preventability of suicide, and reframing stigma. Social norms were targeted through BCTs like credible sources, such as credible role models, to challenge masculine stereotypes and normalize help-seeking. Perceived behavioral control was enhanced by including BCTs such as information about available services and skills training (e.g., conversation starters, emotional vocabulary) to build self-efficacy and to reduce perceived barriers.

This systematic mapping ensured that each element of the training not only addressed a specific TPB construct but also utilized evidence-based BCTs extracted from the previous systematic reviews, which had been shown to influence intentions and behaviors related to mental health help-seeking and stigma reduction. By explicitly linking theory, identified barriers, and BCTs to program content, the intervention was designed to maximize its effectiveness in shifting attitudes, changing social norms, and increasing perceived control, ultimately supporting behavioral change in help-seeking and stigma reduction among male construction workers in Ireland.

### 3.2. Intervention Components

The training was structured into four main sections: (1) Introduction, (2) The Facts, (3) Why Help?, and (4) What I Can Do. Each section was designed to address specific mental health and suicidality-related issues identified as relevant among construction workers in Ireland.

Section (1): Introduction

This section provides an overview of the training, familiarizes participants with the topic, and signposts what they could expect during the session.

Section (2): The Facts

This section explains the rationale for focusing on suicide within the construction industry, including the unique challenges and stressors that impact construction workers’ mental health.

Section (3): Why Help?

This section explores both help-offering and help-seeking behaviors. It dispels common myths surrounding suicide and illustrates the significant and potentially transformative impact that offering or seeking help can have on someone’s life. This section also explores components of positive masculinity that bolster and reinforce help-seeking and help-offering intentions.

Section (4): What I Can Do

This final section provides practical tips on how to engage with someone in distress, where to access appropriate supports, and highlights further suicide prevention training opportunities.

All training components are detailed in Table 1, and a full description of the intervention using the Template for Intervention Description and Replication (TIDieR) checklist [40] is provided in Table 2.

### 3.3. Validating the Delivered Training

To assess the effectiveness of the intervention, a set of validated measurement tools will be employed. These will include the Literacy of Suicide Scale (LOSS) to measure mental health and suicide-related knowledge; the Stigma of Suicide Scale (SOSS) to assess attitudes and stigma related to suicide; and the General Help-Seeking Questionnaire (GHSQ) to measure help-seeking intentions. These findings will inform the direction of any further refinements to the next iteration of the CAIRDE General Awareness Training program.

## 4. Discussion

This paper outlines the systematic development of a theory-informed, stakeholder co-designed mental health intervention for construction workers. The intervention successfully integrates theoretical models with lived experience insights, producing a tailored package designed to address key barriers to accessing mental health support in the construction industry. Applying the Theory of Planned Behavior (TPB) provided a structured framework for understanding the cognitive and social determinants of help-seeking and intervention behaviors. Negative attitudes toward help-seeking, deeply influenced by cultural expectations and masculinity norms within the industry, emerged as a primary challenge. Subjective norms, shaped by workplace culture and prevailing social expectations within the industry, reinforced avoidant behaviors yet also indicated points of intervention through relatable points of contact and peer influence. Perceived behavioral control was constrained by structural and psychological barriers, such as concerns over confidentiality, job security, and service accessibility. Efforts to mitigate these constraints have an important role to play in enhancing help-seeking behaviors across the industry.

Utilizing the TPB for intervention development provided a platform on which to systematically address the aims and core components of the training. Stigma around mental health and suicidality in the construction industry was addressed through the targeting of attitudes and subjective norms among construction workers in key areas of influence, which they themselves helped to identify. The focus on perceived behavioral controls provided a structure on which to highlight key barriers to behavior change and implement strategies to empower individuals to act despite such barriers and/or to help break down some of these barriers in the process. Although there are limited mental health interventions targeting mental health literacy and/or stigma that have integrated the TPB into intervention development, those that have advocated for its effectiveness in this area [40,41]. While many interventions have been developed without explicitly using the TPB, more successful programs have been found to align with its constructs [31].

The study’s findings highlight a number of implications for suicide prevention and mental health intervention development within the construction sector. Targeted workplace interventions should prioritize shifting entrenched social norms and provide accessibility to mental health resources; findings which are consistent with a broad international evidence base [30,42,43,44]. Shifting social norms can best be achieved through the destigmatisation of mental health and suicide, through psychoeducation initiatives delivered by a credible source [31]. Additionally, liaising with local suicide prevention and mental health resources may provide more accessible and tailored resources for construction workers in that area.

This study stands both in contrast to and on the shoulders of previous mental health and suicide prevention trainings developed for the construction industry. The training has been developed with theoretical underpinnings and developed with and for construction workers to function within the specific context and cultural norms of the construction industry. Although previous trainings have been developed, the lack of theoretical underpinnings has limited the exploration of the mechanisms of change used within them. The study and intervention development look to amend this and allow for a transparent, theoretically grounded approach.

Findings from this study support the integration of mental health awareness training into pre-existing safety certification programs. For example, embedding mental health training into mandatory existing training, such as the FÁS Safe Pass Health and Safety Awareness Training Program in Ireland, could help normalize and mainstream conversations on mental health within the industry by capitalizing on established industry infrastructures to do so. Embedding mental health discourse within routine workplace safety protocols has the potential to reinforce sustained cultural transformation by setting the expectation from the start that mental health matters, as many of these mandatory trainings are conducted before one can set foot on the site. Ensuring access to confidential and easily accessible mental health resources remains a critical priority. Enhancing visibility through on-site crisis helplines, designated mental health ambassadors, and anonymous support options may help reduce some of the systemic barriers identified in this study. Concerns about privacy and confidentiality can be addressed through education on how mental health support works and efforts to destigmatize seeking help.

This study had a number of limitations. Due to time constraints, only four co-design workshops were conducted with the construction industry. It is acknowledged that a lengthier consultation and co-design process would have been preferable. Additionally, the training may have benefited from being facilitated by trained construction workers who could serve as a more credible source rather than utilizing outside professional facilitators. Whilst the training of construction workers to facilitate mental health and suicide prevention training was not feasible at this time, this is a key objective in the future development of the program. This will be particularly important in ensuring that, in any future scale-up of the program, it reaches smaller as well as larger construction companies. Future research is needed to explore the feasibility and effectiveness of training provided by construction workers themselves, and to capture differences in facilitation methods and styles.

## 5. Conclusions

This study captures the systematic and iterative development process used in the creation of a general awareness training program targeting mental health literacy, stigma reduction, and help-seeking behaviors among construction workers in Ireland. By leveraging strategic psychoeducation, peer support networks, and embedded workplace interventions, the industry can cultivate an environment that prioritizes mental health and well-being. Future research and policy development should focus on building upon and reinforcing these cultural shifts through evidence-based intervention strategies that facilitate sustainable behavioral change within high-risk occupational environments.

This study extends the application of TPB within occupational mental health research by exploring how industry-specific sociocultural norms influence attitudes, subjective norms, and perceived behavioral control within help-seeking and help-offering contexts. The TPB effectively provides a psychological framework for understanding why people engage in specific behaviors and identifies the factors influencing mental health help-seeking/offering behaviors. The co-design process ensures that the intervention is developed with and for the target audience, integrating the behavioral drivers and associated psychological factors with practical, relevant, and actionable strategies that work within a given socio-cultural context. This study shows the utility of developing a mental health and suicide prevention training using both theoretical underpinnings, such as the TPB, and a person-centered approach, such as co-design, to ensure the acceptability and feasibility of such a training for men. The next step in the development of this intervention includes the pilot delivery of the training, followed by a larger-scale randomized controlled trial of the training.

## Figures and Tables

**Table 1:** Program Theory.

Theory of Planned Behavior	Co-Design Section	Key Uncertainties and Issues to be Addressed	Training Content Post Co-Design Workshop 1	Finalized Content of Training Post Co-Design Workshop 2
**Attitudes**—a person’s favorable or unfavorable evaluation of the problem (mental health) and/or behavior (help-seeking)	Perception about the Problem		1.1 Introduction	1.1 Introduction
**Attitudes**—a person’s favorable or unfavorable evaluation of the problem (mental health) and/or behavior (help-seeking)	Perception about the Problem	**Illness recognition** Unaware that a problem exists (e.g., suicidal behavior, mental illness) Unaware of contributing factors to mental ill-health and suicidal behavior	2.1. Suicide among men 2.2. Suicide and mental health issues among construction workers 2.3. Causes and risk factors of suicide among construction workers	2.1. Suicide among men 2.2. Suicide and mental health issues among construction workers 2.3 Impact of Suicide 2.4. Causes and risk factors of suicide among construction workers
		**Perceived susceptibility** Men not seeing themselves as susceptible to health issues	2.2. Suicide and mental health issues among construction workers	2.2. Suicide and mental health issues among construction workers
		**Recognition of symptoms** Lack of knowledge of symptoms Difficulty recognizing symptoms in oneself	4.1. Know the signs	4.3. Know the signs
		**Perceived severity of symptoms** Low perceived illness severity for symptoms Symptom minimizing Rationalizing symptoms	2.4. The ‘Big Build’	4.3. Know the signs
		**Beliefs about preventability** Belief that suicide is not preventable Belief that people who want to die by suicide cannot be helped	3.1. Preventability of suicide—Dispelling myths	3.1. Preventability of suicide—Dispelling myths
		**Personal stigma of suicide** Suicide seen as a crime/sin. Reluctance to engage with people who are suicidal	3.1. Preventability of suicide—Dispelling myths	3.1. Preventability of suicide—Dispelling myths
	Perception about Support	**Evaluation of the helper** Belief that helpers will not listen, understand, or provide enough time Talked about the helper’s technical competence. Belief that the support environment is unapproachable	3.2. Value, need, and importance of timely help	3.2. Value, need, and importance of timely help
		**Perceived value of help** Belief that seeking support will help address the problem	3.2. Value, need, and importance of timely help	3.2. Value, need, and importance of timely help
		**Perceived need for help** Tendency to wait until symptoms are severe before seeking help	3.2. Value, need, and importance of timely help	3.2. Value, need, and importance of timely help
		**Openness/willingness to accept help**	3.3. Challenging stigma of suicide and help-seeking (reframing)	3.4. Challenging stigma around suicide and help-seeking (reframing)
		**Knowledge of available supports** Lack of awareness about support services, access, and their roles	4.2. Know the available services	4.2. Know the available services and support pathways
	Other	**Self-stigma** Shame, self-blame, social inadequacy, feelings of weakness, embarrassment	3.3. Challenging stigma around suicide and help-seeking (reframing)	3.3. Challenging stigma around suicide and help-seeking (reframing)
		**Pros/cons of disclosure** Fear of negative work-related consequences from disclosing mental health issues		3.2. Value, need, and importance of timely help
		**Ability to communicate emotions** Limited emotional vocabulary and difficulty expressing feelings		3.3 Providing examples of conversation starters and emotional language
**Social norms**—a person’s beliefs about whether others approve or disapprove of help-seeking for mental health. Consists of a person’s evaluation of normative beliefs and their motivation to comply with others.	Willingness to communicate	**Social norms—masculinities, mental health, and help-seeking** Belief that others think men should not have mental health problems Conformity to masculine beliefs about emotional control and self-reliance	3.3. Challenging stigma around suicide and help-seeking (reframing)	3.4. Challenging stigma around suicide and help-seeking (reframing)
**Perceived behavioral control**—a person’s perceived resources, skills, and opportunities to seek help for mental health problems.	Self-efficacy	Confidence to seek help	Addressed throughout training	Addressed throughout training
	Controllability	Perceived control over behavior	4.2. Know the available services	4.2. Know the available services
		Physical barriers (time, cost, availability)	Not addressed	3.5 What are the options
		Physical opportunities	4.2. Know the available services	4.2. Know the available services

**Table 2:** TIDieR Checklist.

Brief Name	
1	General Awareness Training (CAIRDE)
**Why**	
2	The training was developed to reduce suicide rates among construction workers in Ireland. It aims to reduce mental health and suicide stigma, improve mental health literacy, and increase intentions to both seek and offer help.
**What**	
3	The training is primarily psychoeducational. The main resources include a 40-page facilitator’s pack and PowerPoint presentations. Three integrated video resources include: (1) a personal story based on a selection of the lived experiences of construction workers, taken from the focus groups and co-design workshops, and portrayed by an actor; (2) interviews showcasing examples of help-offering within the construction industry; and (3) information on resources and supports available from General Practitioners (GPs) and other mental health support services. Participants also receive wallet cards developed by the National Office for Suicide Prevention (NOSP), which list contact details for support services for those experiencing mental health difficulties or a mental health crisis.
4	Participants are gathered in a meeting room and introduced to the training. It starts with an ice-breaker about participants’ professions, followed by the main content. An interactive “true or false” segment on mental health and suicide myths is included. At the end, support resources are shared along with the NOSP wallet cards.
**Who Provided**	
5	The training is delivered by a professional mental health training facilitator from the Health Service Executive (HSE).
**How**	
6	The training is conducted in person, face to face, in sessions lasting approximately one hour, with up to 30 participants per session.
**Where**	
7	The training is delivered at construction sites, typically in induction training rooms equipped with a projector or large display.
**When and How Much**	
8	The training is a once-off, hour-long session provided free of charge.
**Tailoring**	
9	The training is specifically designed for the construction industry in Ireland, incorporating culturally relevant references and appropriate language.
**How Well**	
10	The training’s effectiveness will be assessed during pilot delivery using the LOSS (Literacy of Suicide Scale), SOSS (Stigma of Suicide Scale), and GHSQ (General Health Seeking Questionnaire) to measure changes in mental health and suicide literacy, stigma, and help-seeking intentions.

## Data Availability

The data presented in this study are available upon reasonable request from the corresponding author due to ethical considerations relating to the sensitive nature of the study.
